# Case Report: Clinical manifestations of retinal detachment in patients with atopic dermatitis: two cases and potential immunopathogenic mechanism

**DOI:** 10.3389/fmed.2026.1887368

**Published:** 2026-09-30

**Authors:** Conghui Zhang, Xin Liao, Hongping Cui, Haiying Jin

**Affiliations:** 1 Department of Ophthalmology, Shanghai East Hospital, School of Medicine, Tongji University, Shanghai, China; 2 Department of Ophthalmology, Shanghai Tenth People’s Hospital, Tongji University, Shanghai, China

**Keywords:** atopic dermatitis, chronic inflammation, ciliary epithelium detachment, eye rubbing, proliferative vitreoretinopathy, retinal detachment, retinal dialysis, retinal tears

## Abstract

**Background:**

Retinal detachment (RD) represents a sight-threatening ocular sequela among individuals affected by atopic dermatitis (AD). Chronic habitual eye rubbing may impair the structural stability of the ocular tissues. Here, we report two patients with AD-associated RD, emphasizing both surgical characteristics and potential immune-mediated mechanisms.

**Methods:**

Two cases of AD-associated RD were included. For each one, the clinical picture, intraoperative findings, and potential immune pathway hypotheses were taken into account.

**Results:**

Our first patient was a 15-year-old boy with a history of toric intraocular lens implantation who initially presented with elevated intraocular pressure and vitreous hemorrhage, later developing RD characterized by retinal dialysis, retinal tear, and ciliary epithelium detachment. Patient two was a man of 43, in whom RD coexisted with posterior subcapsular cataract as well as astigmatism. Severe systemic AD was present in both, and both had a long history of rubbing their eyes. Each underwent vitrectomy with internal tamponade. In one of them, recurrence ensued because of proliferative vitreoretinopathy.

**Conclusion:**

This series illustrates the typical features and surgical challenges of RD in AD, which arise from chronic eye rubbing-induced mechanical damage and astigmatism. Timely ophthalmic evaluation and systemic AD control aid visual preservation. Our findings propose a preliminary hypothesis of interactive immune and mechanical mechanisms underlying AD-associated RD, which warrants further experimental validation.

## Introduction

A chronic inflammatory disease of the skin, atopic dermatitis (AD) presents clinically with relapsing eczematous lesions, a defective epidermal barrier, and an immune profile. Diagnosis is mostly clinical. The criteria call for three or more major features (itching, a typical lesion morphology and distribution, a chronic or relapsing course, and personal or familial atopy) plus three of the minor features. Routinely employed in clinical practice, this framework helps to identify those at heightened risk of both systemic and ophthalmic involvement. Beyond the skin itself, AD affects the eye in a number of ways, ranging from recurrent conjunctivitis and Dennie–Morgan infraorbital folds to keratoconus, anterior subcapsular lens changes, lid dermatitis, and periocular eczema. Balyeat first reported on the association of retinal detachment (RD) with AD as early as 1937 (1). The overall prevalence of RD in patients with AD ranges from 4% to 8%, and the risk is markedly elevated in those with severe AD (2). Onset of disease usually occurs in the second or third decade of life, and involvement is commonly bilateral. Published series have been drawn predominantly from East Asia, where prevalence in those with severe disease has been estimated at roughly 11.4% (3). About half of the affected eyes also harbor a subcapsular cataract. Warning symptoms such as worsening floaters or photopsia are frequent (4). The pattern of AD-associated RD is fairly stereotyped: the temporal periphery is preferentially affected, retinal dialysis and giant retinal tears (GRT) are common, and recurrence after surgery is high. Intraocular lens (IOL) dislocation is also frequent and contributes to the generally poor visual outcome.

Mechanical injury, however, is not the only factor at work. AD is now seen as a Th2-skewed chronic inflammatory disorder in which immune dysregulation and a defective epithelial barrier go hand in hand. The IL-4/IL-13 cytokine pair, signaling via the JAK-STAT axis, plays a pivotal role here, fueling fibroblast activation, extracellular-matrix remodeling, and a persistent inflammatory state (5–7). Over time, persistent exposure to these cytokines may reshape the mechanical behavior of ocular connective tissue, which in turn could predispose the retina to tractional forces and to structural compromise (8, 9). When the additional roles of frequent eye rubbing and ongoing periorbital inflammation are taken into account, a plausible account is offered for the unique array of ocular sequelae seen in patients with AD. From a translational immunology perspective, AD serves as a valuable model for investigating how chronic Th2-driven inflammation interacts with tissue biomechanics, with potential implications for understanding immune–stromal interactions in other inflammatory diseases.

## Case presentations

### Case1

A boy aged 15 was referred for sudden blurring of vision in the right eye, which he had first noticed on the day before. Around seven months earlier, he had received bilateral phacoemulsification with toric IOL implantation for dense cataract together with corneal astigmatism. Refractive values were −2.87 D on the right and −3.74 D on the left side (Figures 1C,D). No detailed cataract grading was documented in the initial medical records. However, intraoperative toric IOL implantation confirmed clinically meaningful corneal astigmatism. This finding indicated atopic cataract secondary to AD. A diagnosis of systemic AD had previously been established by a dermatology specialist in accordance with the Hanifin–Rajka diagnostic criteria (Figures 1A,B). He denied any history of ocular trauma, although periocular eczema was clearly visible on examination (Figures 1A,B).

**Figure 1:**
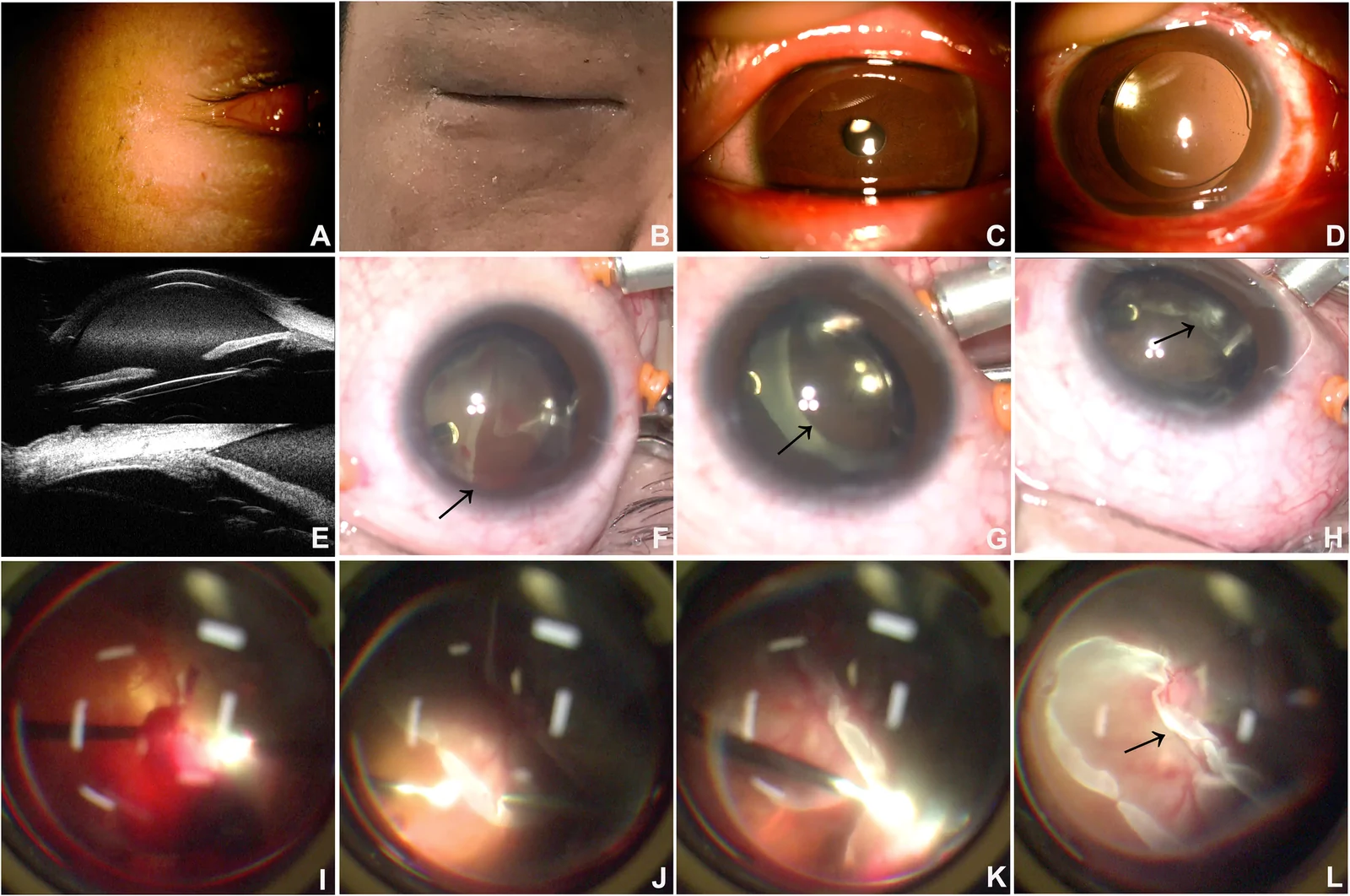
Clinical features of case 1. **(A,B)** Periocular eczema. **(C,D)** Toric intraocular lenses. **(E)** UBM demonstrates diffuse punctate hyperreflective deposits within the anterior chamber, with no angle crowding or peripheral anterior synechiae. **(F–L)** Intraoperative findings: arrows in **(F,G)** show retinal dialysis; the arrow in **(H)** indicates ciliary epithelial detachment; the arrow in **(L)** denotes a giant retinal tear running parallel to the ora serrata (6:30–11:00).

On first review, best-corrected visual acuity (BCVA) was 20/20 bilaterally, while the right-sided IOP had risen to 34 mmHg. On slit-lamp examination there was mild edema of the cornea, with KPs of a pigmented type and blood-tinged opacity within the anterior chamber. The implanted lens remained centrally positioned, but vitreous opacities prevented adequate fundus visualization. Ultrasound biomicroscopy (UBM) revealed widely patent angles along with hyperreflective material in the anterior segment (Figure 1E). Such deposits were observed as diffusely scattered fine punctate floating echoes; neither crowding of the angle nor peripheral anterior synechiae was apparent. There were no irregularities of the iris configuration, and the ciliary body appeared normal. These findings raised an initial suspicion of uveitis–glaucoma–hyphema (UGH) syndrome; nevertheless, central placement of the IOL within the capsular bag was confirmed. Aqueous humor sampling was not performed because markedly elevated IOP and dense vitreous hemorrhage precluded safe anterior chamber paracentesis.

Three days of pharmacologic treatment led to normalization of IOP, with concomitant clearing of the anterior chamber. One week later, however, sudden and profound loss of vision ensued. The pressure had fallen to 8.2 mmHg, while dense hemorrhage into the vitreous became apparent.

Ultrasonography (B-scan) confirmed RD together with vitreous hemorrhage, which prompted pars plana vitrectomy (PPV). The patient underwent 23-gauge PPV without scleral buckling due to severe preoperative vitreous hemorrhage (Figure 1I). Complete intraoperative posterior vitreous detachment (PVD) was performed, followed by extensive vitreous base shaving up to the ora serrata to fully eliminate peripheral vitreoretinal traction. The retina was flattened using perfluorocarbon liquid, and circumferential endolaser retinopexy was applied to seal all retinal dialysis. The toric intraocular lens remained well-centered within the capsular bag throughout the procedure. Given the extensive retinal dialysis requiring durable mechanical support, silicone oil tamponade was selected for long-term retinal stabilization. At surgery, an extensive dialysis at the ora serrata extending from the 5:30 position around to 11:00 was uncovered (Figures 1F,G,J,K). Running parallel to the serrated margin, a giant retinal tear (6:30–11:00, ≥90°) engaging the vascular arcades was likewise documented (Figure 1L). Inferotemporal detachment of the ciliary epithelium was, in addition, identified while peripheral vitrectomy was performed (Figure 1H). By definition, ora serrata defines the transition into the ciliary body of the retina, marking its anterior boundary. Firm adhesion of the vitreous base is established at and just behind this border, dialysis at the ora serrata denotes a break occurring at this junction, namely a disinsertion of the retinal tissue within the vitreous base. A giant retinal tear, by contrast, is a full-thickness defect spanning ≥90° of the circumference and positioned behind the vitreous base. Ciliary epithelium detachment refers to separation of the ciliary epithelium from the ciliary stroma, which occurs when severe RD extends beyond the ora serrata and tears the peripheral pigment epithelial layer. Under intraoperative microscopy, we distinguished it from other anterior peripheral pathologies by typical morphological manifestations of detached ciliary epithelium. For internal tamponade, silicone oil was instilled, with removal completed three months thereafter (Figures 2A,B).

**Figure 2:**
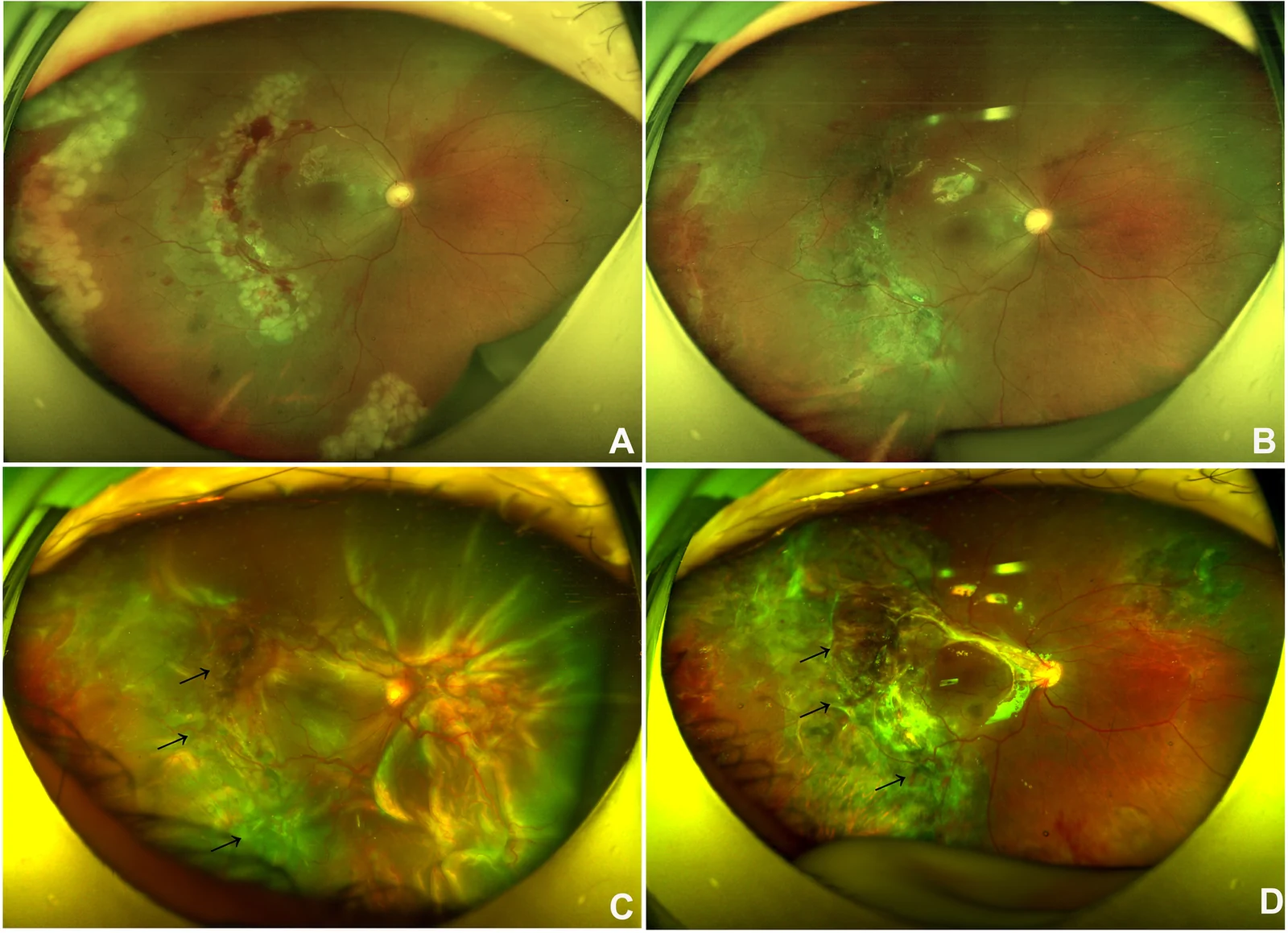
Postoperative and recurrent retinal findings. **(A,B)** Postoperative fundus photos show retinal dialysis, retinal tear parallel to the ora serrated with silicone oil tamponade. **(C)** Recurrent retinal detachment; arrows denote vitreoretinal traction secondary to proliferative vitreoretinopathy after oil removal. **(D)** Postoperative fundus photo; the arrow indicates retinal scarring.

After surgery, BCVA recovered to 20/33; the patient received counseling to refrain from rubbing his eyes and to maintain active dermatologic therapy targeting his AD. Despite this, redetachment manifested one month following oil removal, accompanied by pronounced proliferative vitreoretinopathy (PVR) (see Figures 2C,D). Repeat PPV with silicone oil tamponade was then carried out. Vision deteriorated, and quantification was not feasible in the oil-filled eye.

### Case2

A 43-year-old man presented with progressive blurred vision and a visual field defect in his left eye that had persisted for approximately one year. AD was diagnosed by dermatologists strictly in accordance with the Hanifin–Rajka diagnostic criteria. He carried a long-standing diagnosis of AD and, at presentation, was receiving oral immunosuppression (Figure 3E). Examination at the slit lamp identified posterior subcapsular cataract (PSC) together with corneal astigmatism, measured at −1.68 D on the right side and −3.21 D on the left (Figures 3A–D).

**Figure 3:**
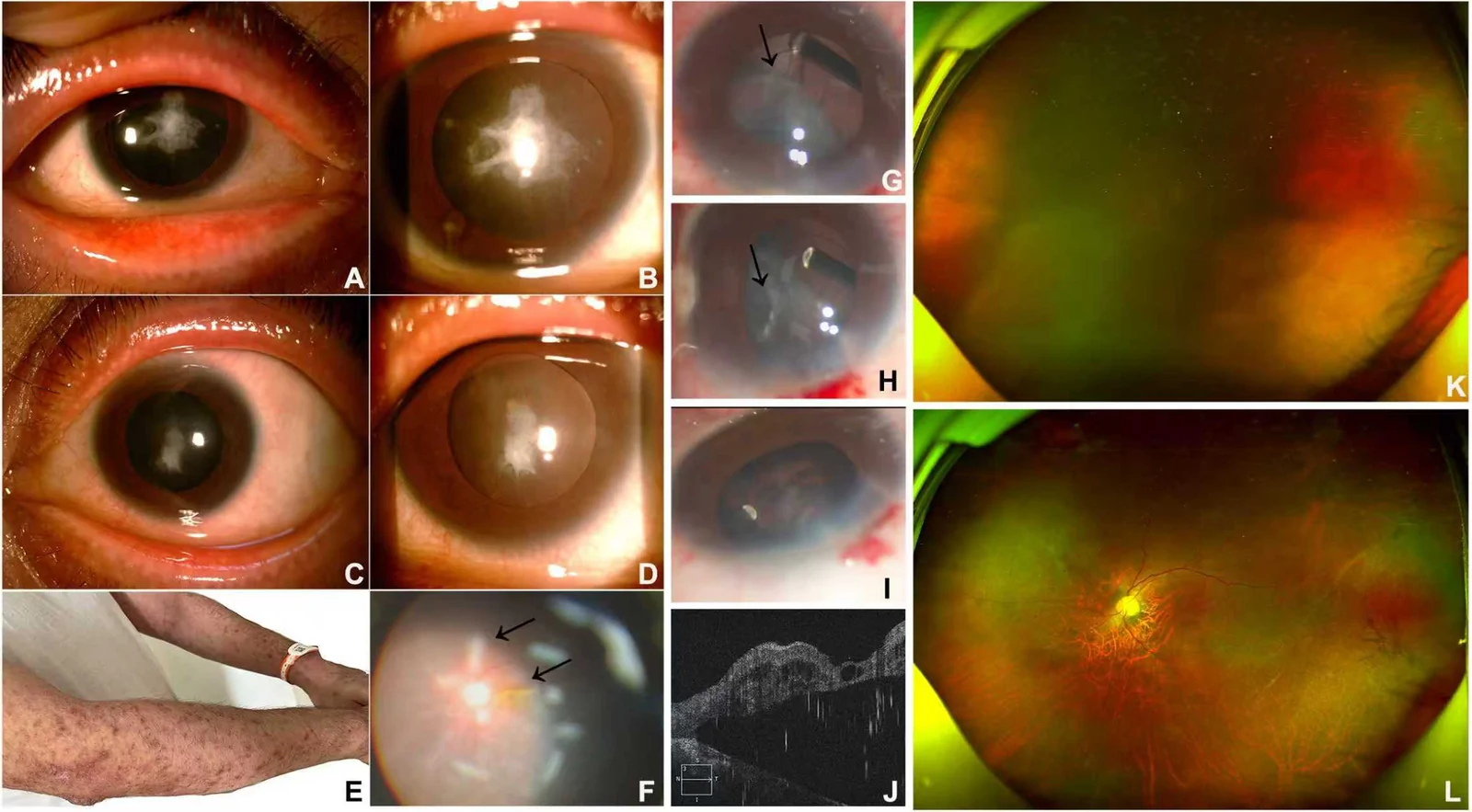
Clinical features of case 2. **(A–D)** Posterior subcapsular cataract. **(E)** Systemic dermatitis. **(F–J)** Intraoperative findings. **(F)** Arrow denotes retinal detachment (3:00–1:00). **(G)** Arrow indicates the retinal break. **(H,I)** Arrows show ciliary epithelial detachment and pigmentation. **(J)** Preoperative OCT. **(K)** Preoperative fundus image: vitreous opacity and retinal detachment. **(L)** Retina remains fully attached at 3-month follow-up.

On fundoscopy, retinal detachment extended between the 3:00–1:00 positions (Figure 3F), with the principal break located at the 12:00 meridian (Figure 3G) (scleral depression resulted in ocular rotation). This patient also received 23-gauge minimally invasive PPV without scleral buckling, based on the patient's age, retinal detachment range, requirement for combined cataract surgery, and the presence of AD-related fragile peripheral retinal tissue. Intraoperative procedures were consistent with those in Case 1, including complete PVD, full-thickness circumferential vitreous base shaving up to the ora serrata, perfluorocarbon liquid-assisted retinal reattachment, and circumferential endolaser retinopexy with routine fluid–air exchange. Considering the superior retinal breaks and relatively mild retinal lesions, C3F8 gas was used for long-acting intraocular tamponade. During the pars plana vitrectomy, ciliary epithelial detachment and abundant RPE (retinal pigment epithelial) cells were noted scattered throughout the vitreous space (Figures 3H,I).

The eye was tamponaded with C3F8. Recovery proceeded uneventfully; over the three-month follow-up window, the retina stayed attached and vision remained stable. Further examinations disclosed neither redetachment nor any new retinal breaks (Figure 3L). The detailed clinical data concerning atopic dermatitis manifestations and treatment profiles of the two cases were shown in Table 1.

**Table 1:** Detailed clinical characteristics of atopic dermatitis-related manifestations and corresponding therapeutic strategies.

Parameter	Case 1	Case 2
Age (years)	15	43
Sex	Male	Male
AD duration (years)	5	15
AD severity grading	Moderate	Severe
Serum IgE concentration	NA	>2,712 g/L
Peripheral eosinophil counts	0.55*10^9/L	0.19*10^9/L
Medication history	Dexamethasone/ tacrolimus Ointment	Dupilumab
Ocular history	Atopic keratoconjunctivitis	Atopic keratoconjunctivitis
Cataract/IOL status	Pseudophakic (Toric IOL)	Posterior subcapsular cataract
Laterality (OD/OS/OU)	OD	OS
Preoperative BCVA (LogMAR)	2	1.3
Preoperative IOP (mmHg)	8.2	8
location of retinal break	5:30–11:00	12:00
Retinal dialysis/giant retinal tear	Retinal dialysis and giant retinal tear	Retinal hole
Intraocular tamponade	Silicone oil	C3F8
Recurrence during follow-up	4 months(1 month after oil removal)	None
Final BCVA (LogMAR)	1	0.3
Follow-up duration (months)	12	3

## Discussion

Pathogenetic links of atopic cataract have been drawn with cutaneous lesions of the face, serum IgE elevations (6, 10), and use of corticosteroid agents. According to Gnana Jothi, surgery for atopic cataract was commonly required between 18 and 31 years of age, with astigmatism being mild. Within that case series, lens dislocation was documented in six eyes from six different patients (11). In our case, the patient was managed with toric IOL implantation in both eyes (Alcon), and BCVA of 20/20 was attained in each eye after the procedure. Of note, three years on the IOLs continued to be well-centered, paralleling effective control of dermatitis and abstention from rubbing the eyes. Comparable observations applied to Case 2, in whom severe PSC was accompanied by corneal astigmatism (−1.68 D OD, −3.21 D OS). Because the risk of lens dislocation is heightened in AD, intraoperative precautions become critical. Such precautions involve fashioning a continuous curvilinear capsulorhexis of suitable diameter, limiting zonular strain during hydrodissection. Where weakness of the zonule is suspected, a capsular tension ring should be considered. Astigmatism and IOL dislocation in AD are probably multifactorial. Contributing factors may include persistent eye rubbing, biomechanical compromise of the cornea or zonule, and inflammation-driven extracellular matrix remodeling (11). Such a view is corroborated by Ahmad M, in whose work the corneal surface asymmetry index was reported to rise from 0.27 up to 0.89 immediately on completion of eye rubbing (12). Salient findings in our first patient included the following: dialysis of retinal tissue adjacent to the ora serrata, a large tear oriented along the serrated margin, inferotemporal separation of the ciliary epithelium, and engagement of vessel-bearing arcades giving rise to hemorrhage within the vitreous. Such findings imply that detachment was initiated through retinal dialysis, instead of arising from a typical retinal break. This configuration mirrors trauma-related RD, which is characteristically situated within the inferotemporal quadrant (13). For clarity, the clinical timeline may be set out chronologically: cataract surgery (seven months earlier), onset of symptoms (one day before presentation), conservative medical therapy at first, vitrectomy for RD, withdrawal of silicone oil (three months after surgery), and the subsequent return of detachment (one month thereafter). The risk of RD following cataract extraction has been reported to rise two- to three-fold (14), and may climb as much as ten-fold among younger individuals (15), this is most plausibly attributable to firmer vitreoretinal attachments that favor traction in the postoperative period. An analogous pathway operates in AD: vitreoretinal traction together with peripheral breaks is promoted by repetitive eye rubbing alongside inflammation, an effect compounded by the elevated frequency of early-onset cataract requiring surgery. Limiting disturbance of the vitreous and zonule is therefore of paramount importance in such patients. To clarify the multifactorial pathogenesis of RD in this pseudophakic patient, we conducted a differential etiologic analysis. First, the uneventful phacoemulsification performed by experienced surgeon involved negligible vitreous and zonular traction. The risk of RD might be increased after cataract surgery in AD patients, but this case was considered AD-associated RD based on its characteristic features, with cataract surgery and chronic eye rubbing as potential triggers (16). Second, traumatic RD was ruled out with no trauma history. Third, UGH syndrome was excluded, as the toric IOL was stably centered in the capsular bag without iris chafing or inflammation. This patient possessed typical AD-related high-risk phenotypes confirmed by recent studies: systemic and periorbital AD, high astigmatism, premature cataract, chronic eye rubbing and inherent ocular fragility. Collectively, the retinal detachment is most plausibly explained by a multifactorial process in which AD-associated ocular fragility and chronic mechanical stress acted as key predisposing factors, with prior cataract surgery and eye rubbing potentially serving as contributory but non-specific risk modifiers, rather than isolated causal factors.

Patients diagnosed with giant retinal tear are frequently complicated by PVR. As reported by Lee and Kim (17). Extensive retinal defects, persistent subretinal fluid and chronic intraocular mild inflammation caused by GRT continuously stimulate cellular proliferation. This leads to rapid progression of PVR from mild or moderate stage to severe stage. Conversely, traction bands and proliferative membranes secondary to PVR further stretch and enlarge the original giant retinal tear. This increases surgical difficulty and postoperative recurrence rate. Patients with systemic inflammatory diseases such as atopic dermatitis may present with higher intraocular inflammatory activity and potentially a significantly higher PVR recurrence rate after GRT surgery. Early pars plana vitrectomy with complete removal of proliferative membranes appears to be key to reducing recurrence. For cases with extensive GRT, severe PVR or recurrent history, silicone oil tamponade is preferred to relieve traction and inhibit proliferation. In AD-associated RD, the principal postoperative complication was recurrence of detachment, and this often translated into poor visual outcomes. In one multicenter retrospective series, the primary reattachment rate stood at 75.3% and rose to 92.6% by the time of final follow-up (18). A Hong Kong series reported worse figures, with rates of 55.6% at primary repair and 77.8% on final assessment (19). Because these patients tend to be young, with an inflamed eye and tight vitreoretinal adhesions, scleral buckling may often be a better choice. It performs well when the breaks are peripheral and the vitreous is intact, whereas pars plana vitrectomy carries a higher risk of iatrogenic breaks, recurrence, and PVR. With the aim of forestalling recurrence, thorough posterior combined with peripheral vitrectomy was undertaken in order to relieve traction at the vitreous base. Silicone oil tamponade was then instituted. These efforts notwithstanding, re-detachment supervened one month after removal of the oil, alongside severe PVR. Among the predisposing factors are dialysis of the retina, giant retinal tears, exposure of RPE, the patient's younger age, and intraocular hemorrhage accompanied by elevated vitreous protein content (5). Where the disease severity is comparable to the present case, extended silicone oil tamponade warrants consideration. Decisions about when to withdraw the oil must weigh not only the anatomic stability of the retina but also the activity of facial AD, since vigorous treatment of cutaneous inflammation has been shown to lower the chance of recurrence (20). In our opinion, scleral buckling could still be a potential first-line therapy for atopic dermatitis-related retinal detachment that meets the surgical indications for external buckling.

Immune dysregulation has emerged as a potential mechanism connecting AD with ocular complications. Previous studies have suggested that zonular instability and lens opacification may be associated with chronic inflammation and exposure to corticosteroid therapy (10, 12, 21). Experimental evidence indicates that activation of the JAK–STAT pathway downstream of IL-4 and IL-13 signaling can induce periostin, matrix metalloproteinases (MMPs), and transforming growth factor-β (TGF-β), factors implicated in extracellular matrix remodeling and tissue fragility (7, 9). Dysregulation of the JAK–STAT cascade has also been implicated in various pathological conditions, including tumor progression and drug resistance, underscoring its broad relevance as a signaling node that integrates inflammatory and proliferative signals (22). These observations suggest a hypothesis that chronic inflammation in AD could create a microenvironment that facilitates profibrotic signaling following retinal detachment (Figure 4). IL-4 and IL-13 exert complex regulatory effects on chemokine secretion in human retinal pigment epithelial (HRPE) cells, monocytes, and their co-culture system, indicating their potential involvement in the treatment of ocular inflammatory and proliferative disorders (23). Notably, monocyte functional specialization is governed by distinct transcriptional programs, which may modulate the intraocular immune microenvironment and influence the progression of proliferative vitreoretinopathy in inflammatory conditions such as AD (24). Previous *in vitro* studies have demonstrated that IL13Rα2 is significantly overexpressed in vitreous-stimulated ARPE-19 cells and clinical PVR epiretinal membranes. Blocking IL13Rα2 substantially suppresses vitreous-mediated RPE contraction, supporting IL-13/IL13Rα2 signaling axis as a potential contributor in PVR pathogenesis (25). Moreover, extensive experimental and pathological evidence has established epithelial-mesenchymal transition (EMT) of RPE cells as a key event in PVR pathogenesis, characterized by enhanced cellular migration, proliferation, extracellular matrix deposition, and membrane contraction. Moreover, oxidative stress and apoptosis of retinal cells have been implicated in the pathogenesis of retinal injury and fibrotic sequelae, as demonstrated by the protective role of TYRP1 against retinal ganglion cell oxidative damage via PMEL binding (26). Based on studies discussed above, we hypothesize that chronic immune dysregulation and persistent IL-4/IL-13 may interact with retinal detachment and RPE activation. This interaction could promote RPE EMT, tissue fibrosis, and subsequent PVR development. Although intraocular cytokine analysis was not conducted in the present study, chronic immune dysregulation may represent one of several factors predisposing susceptible patients to PVR development after retinal detachment repair (Figure 4). This model is proposed as a mechanistic hypothesis rather than a definitive causal conclusion. Because the present hypothesis remains preliminary, experimental confirmation of these pathways is warranted.

**Figure 4:**
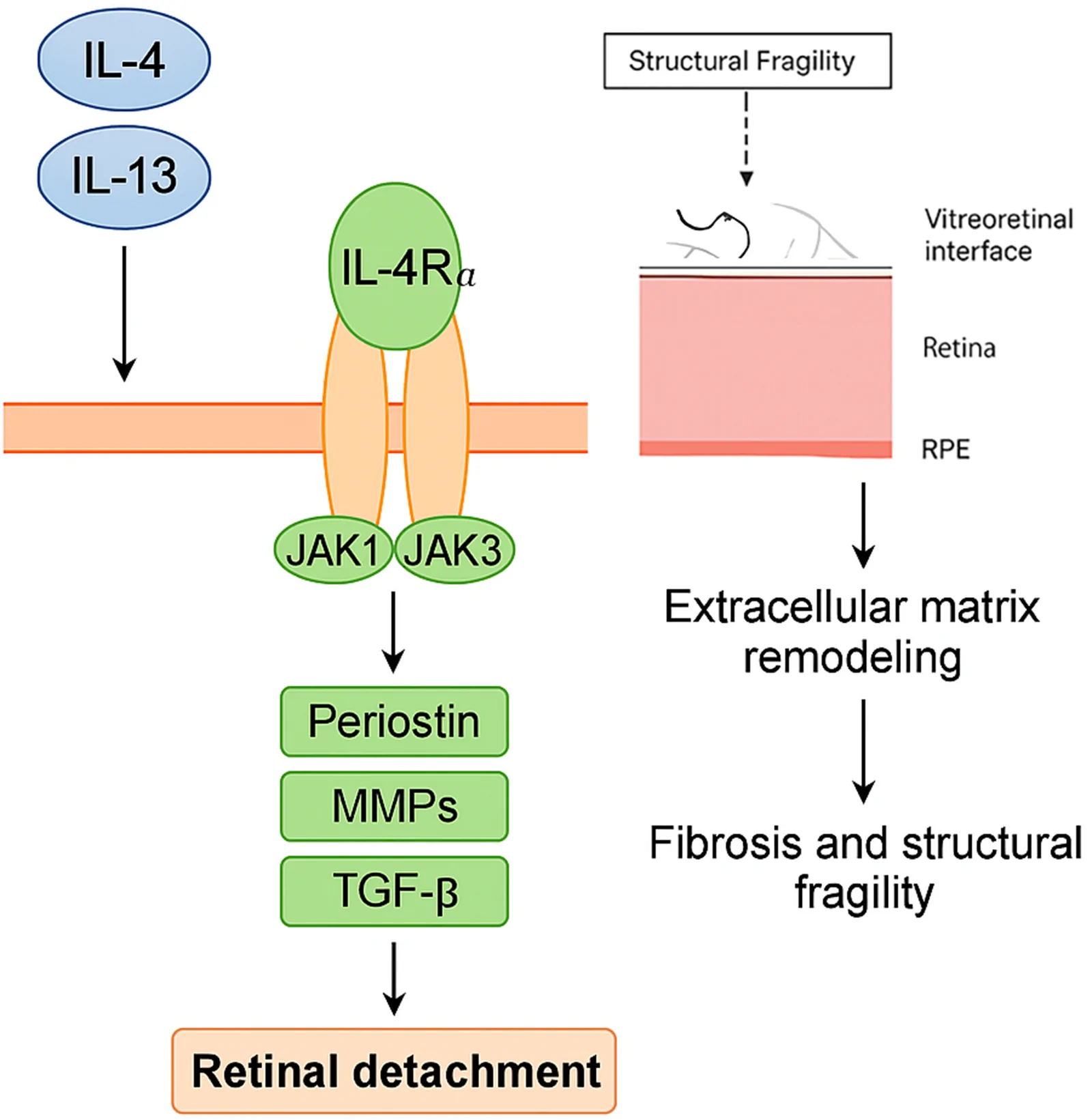
Schematic illustration of a hypothetical immunopathogenic model. This diagram depicts a speculative pathway in which chronic Th2-type inflammation (IL-4/IL-13) may activate JAK–STAT signaling, leading to downstream effects including extracellular matrix remodeling (periostin, MMPs, TGF-β), RPE activation/EMT, and ultimately fibrotic changes such as PVR.

A joint consensus document addressing dupilumab-induced ocular surface conditions (DROSD) has been produced through collaboration between dermatology and ophthalmology professional bodies in the UK; this document delineates strategies for ocular adverse reactions, age-tailored approaches, and referral indications in AD, and its applicability extends to ocular surface disease arising in association with tralokinumab as well as lebrikizumab (27). The patient in case 1 received topical dexamethasone combined with tacrolimus ointment, the patient in Case 2 was treated with dupilumab. Cutaneous symptoms improved in both patients after treatment. However, given the potential for dupilumab to paradoxically induce ocular surface inflammation, interdisciplinary coordination between dermatologists and ophthalmologists remains essential for optimal management of AD patients with ocular comorbidities.

## Conclusion

The present case series underscores the surgical difficulties and therapeutic considerations encountered when AD is complicated by both cataract and RD. AD-associated retinal detachment presents typical features including retinal dialysis, ora serrata-parallel retinal tears, and ciliary epithelium detachment. Chronic eye rubbing in AD patients appears to contribute to mechanical ocular injury, leading to significant astigmatism and retinal detachment; these findings are consistent with previous reports. Timely ophthalmic evaluation and systemic AD control are recommended for visual preservation. From a translational perspective, this case series highlights a potential interplay between chronic immune dysregulation and mechanical trauma in AD-associated RD pathogenesis. The proposed mechanistic model—centered on IL-4/IL-13/JAK-STAT signaling, RPE EMT, and fibrotic progression—is speculative and intended to generate hypotheses for future investigations. This study has several limitations. First, the small sample size (two cases) precludes generalization of our findings to the broader AD population. Second, intraocular cytokine analysis was not performed, limiting our ability to directly validate the proposed immunopathogenic hypothesis involving IL-4/IL-13 signaling in PVR pathogenesis. Third, follow-up duration was uneven between the two cases (12 months in Case 1 versus 3 months in Case 2), which constrains comparative assessment of long-term surgical outcomes and recurrence risk. Fourth, the retrospective nature of this case series introduces inherent selection bias. These limitations notwithstanding, our findings provide a mechanistic hypothesis that warrants confirmation. Prospective studies incorporating aqueous/vitreous cytokine profiling and standardized longitudinal follow-up protocols.

Clinical recommendations:

- Hallmark features of AD-associated RD encompass retinal dialysis, a tear in the retina running along the serrated margin, and detachment of the ciliary epithelium.
- Among AD patients, persistent eye rubbing may precipitate early-onset cataract, considerable astigmatism, together with retinal detachment.
- For RD occurring in atopic dermatitis, prompt ophthalmologic assessment alongside systemic immunologic control is indispensable for preserving vision.

## Data Availability

The original contributions presented in the study are included in the article/Supplementary Material, further inquiries can be directed to the corresponding author.
